# Generalized seizures and transient contralateral hemiparesis following retrobulbar anesthesia: a case report

**DOI:** 10.1186/s12871-015-0084-y

**Published:** 2015-07-28

**Authors:** Maria Dettoraki, Chrisafoula Dimitropoulou, Nikolaos Nomikarios, Marilita M Moschos, Dimitrios Brοuzas

**Affiliations:** 1 1st Department of Ophthalmology, University of Athens, “G. Gennimatas” General Hospital of Athens, 10 G. Papandreou Str, Byron Athens, 16231 Greece; 2 Department of Anesthesiology, “G. Gennimatas” General Hospital of Athens, Athens, Greece

**Keywords:** Seizures, Hemiparesis, Retrobulbar anesthesia

## Abstract

**Background:**

Retrobulbar block is a local anesthetic technique widely used for intraocular surgery. Although retrobulbar anesthesia is considered to be relatively safe, a number of serious adverse events have been reported. To our knowledge, immediate onset of generalized seizures with contralateral hemiparesis after retrobulbar anesthesia has not been reported.

**Case presentation:**

A 62-year-old Caucasian healthy male with a right eye retinal detachment was admitted for pars plana vitrectomy. During retrobulbar anesthesia with ropivacaine and before needle withdrawal, the patient developed twitching of the face which rapidly progressed to generalized tonic-clonic seizures. Arterial oxygen saturation decreased to 75 %. Chin lift was performed and 100 % oxygen was administrated via face mask, which increased saturation to 99 %. Midazolam 2 mg was administrated intravenously to control seizures. After cessation of seizures, left-sided hemiparesis was evident. Brain computed tomography and electroencephalogram were normal 3 h later. The patient underwent pars plana vitrectomy under general anesthesia 4 days later.

**Conclusion:**

Serious complications of local anesthesia for ophthalmic surgery are uncommon. We present a case in which generalized tonic-clonic seizures developed during retrobulbar anesthesia, followed by transient contralateral hemiparesis. The early onset of seizures indicated intra-arterial injection of the anesthetic. Our case suggested the need for close monitoring during the performance of retrobulbar anesthesia and the presence of well-trained personnel for early recognition and immediate management of the complications.

## Background

Retrobulbar block is a local anesthetic technique widely used for intraocular surgery. Although retrobulbar anesthesia is considered to be effective and relatively safe [1], some serious sight and life-threatening adverse events have been reported. Cases of globe perforation, retrobulbar hemorrhage and optic nerve injury have been presented [2]. Life-threatening complications include myocardial ischaemia, cardiovascular depression, respiratory arrest and convulsions [1–4]. In most cases, the exact mechanism of local anesthetic spread to brain structures is not fully understood.

We present a case in which generalized tonic-clonic seizures developed during retrobulbar anesthesia with ropivacaine, followed by transient contralateral hemiparesis and we discuss the possible mechanisms of central nervous system spread of the anesthetic agent. To our knowledge, immediate onset of generalized seizures with contralateral hemiparesis after retrobulbar anesthesia has not been reported.

## Case presentation

A 62-year-old Caucasian male (height 1.65 m, weight 65 kg and American Society of Anesthesiologists score 1) with a right eye retinal detachment was admitted for pars plana vitrectomy under local anesthesia. The past medical history was unremarkable and the physical examination, electrocardiogram and laboratory studies were normal. There was no history of epilepsy or allergy.

Preoperatively, the blood pressure (BP) was 125/80 mmHg, the heart rate (HR) was 80 beats/minute and the arterial oxygen saturation (SpO2) was 99 %. A peripheral venous catheter 20 g was inserted in the left hand and powered by an infusion of normal saline (0.9 %). The patient did not receive any medications before the application of local anesthesia except for tropicamide 0.5 % and cyclopentolate 1 % eye drops instilled in the right eye (one drop every 5 minutes × three times). The periocular skin, eyelids and lashes were cleaned with 10 % povidone iodine and the anesthetic technique was explained to the patient. A retrobulbar anesthesia with the patient’s eye in primary gaze position was given by the surgeon. The anesthetic ropivacaine hydrochloride 7.5 mg/ml was administrated and a 23GA, 38 mm, 1½ inch, blunt-tipped needle (Alcon, Central Texas Medical Equipment and Supplies, USA) was used. The injection was performed in the lower temporal retrobulbar space with 6 ml of ropivacaine.

Before needle withdrawal and completion of anesthetic injection, the patient developed twitching of the face which rapidly progressed to generalized tonic-clonic seizures. The needle was immediately removed. Arterial oxygen saturation (SpO_2_) decreased to 75 %. Chin lift was performed and 100 % oxygen was administrated via face mask, which increased saturation to 99 %. Blood pressure (BP) increased to 140/90 mmHg and heart rate (HR) was 93 beats/minute. Midazolam 2 mg was administrated intravenously by the anesthesiologist about 4 min later and controlled the seizures immediately. After cessation of seizures, left-sided hemiparesis was evident from the asymmetric reaction to painful stimulation and gravity (no visible muscle contraction of the affected side). The neurological examination by specialist a few minutes later was normal. The patient was alert and oriented, could follow commands and no signs of contralateral hemiparesis were detected. Surgery was postponed. Brain computed tomography and electroencephalogram were normal 3 h later. The patient underwent pars plana vitrectomy under general anesthesia 4 days later.

## Discussion

A rare but serious systemic complication related to retrobulbar anesthesia is generalized seizures. The incidence of central nervous system complications in a series of 6000 retrobulbar blocks was 1 in 375 [1], while in another study the incidence of systemic adverse events associated with local anesthesia for intraocular surgery was 0.9 % [2].

Several mechanisms may lead to central nervous system spread of the local anesthetic agent during retrobulbar block. One mechanism is the inadvertent injection of the anesthetic solution in the ophthalmic artery or its branches. Central nervous system toxic responses can result from retrograde passage of a local anesthetic injected under pressure from the ophthalmic artery to internal carotid artery and delivery to the thalamus and other midbrain structures [5–7]. The ophthalmic artery usually runs above the optic nerve but in 15 % of patients it lies inferior to the optic nerve in the orbital cavity [8], where retrobulbar anesthesia is usually performed. Clinical signs vary from convulsions to cardiopulmonary arrest and the onset is rapid [1, 9, 10].

Another possible mechanism leading to central nervous system intoxication is the unintentional injection of anesthetic in the optic nerve sheath and entry into the subdural or subarachnoid space [11, 12]. The onset of symptoms range from 2 to 40 min after the retrobulbar anesthesia in most cases [1]. The use of long needles of 31 mm or more [1, 13] or an uncooperative patient unable to keep the eyes in primary gaze position during the injection increases the risk of the optic nerve puncture.

Our patient had no history of allergy, epilepsy, alcoholism, was not on any medication and also all the biochemical investigations pro- and after the incidence were normal. Therefore, no causative factor which may potentiated seizures [14] was detected. Furthermore, brain computed tomography and electroencephalogram were normal, eliminating the possibility of a brain damage or stroke. It is also unlikely that the seizures and hemiparesis were caused by an accidental intravenous injection of the anesthetic during retrobulbar block, since the total dose of local anesthetic used in our case was less than the intravenous toxic dose described to cause systemic toxicity [15, 16].

In our case, generalized tonic-clonic seizures developed during retrobulbar block before withdrawal of the needle and completion of the injection of the anesthetic agent. Using a 38 mm long needle, we cannot exclude the central spread of local anesthetic through an accidental puncture of the optic nerve sheath. Although a blunt needle might be less likely to penetrate the optic nerve sheath, there is a report of unintentional injection of local anesthetic in subarachnoid space with this type of needle [11]. However, in our patient, the omission of needle aspiration test before the injection and the immediate onset of seizures (before needle withdrawal) are in favor of the inadvertent intra-arterial injection of ropivacaine in the ophthalmic artery or its branches with retrograde flow into the internal carotid artery and delivery to brain structures.

A variable onset of neurological signs after retrobulbar or peribulbar anesthesia has been reported, ranging from the time of needle withdrawal [9] to 40 min later [1]. Meyers *et al.* [9] presented two cases in which grand mal seizures with respiratory obstruction developed immediately after retrobulbar injection of 3 ml of lidocaine with epinephrine in the first case and 5 ml of a solution contained bupivacaine, lidocaine and epinephrine in the second case. Bensghir *et al.* [17] reported a case in which generalized convulsions occurred 6 min following peribulbar injection of a mixture contained lidocaine and bupivacaine. In our case, 6 ml of ropivacaine were injected in the inferior temporal retrobulbar space which immediately initiated generalized seizures.

Several anesthetics responsible for central nervous system toxicity following local anesthesia for intraocular surgery have been reported; among them bupivacaine was the most frequently associated with seizures [18]. Ropivacaine is considered to cause less central nervous system and cardiac toxicity than bupivacaine [15, 16, 18] and constitutes an effective alternative to bupivacaine [19]. Pragt *et al.* [12] in 2006 reported one patient with localized convulsions and brief contralateral hemiparesis 9 min after retrobulbar injection of 5 ml 1 % ropivacaine. The authors could not determine the exact cause of convulsions and hemiparesis but suspected the subarachnoid injection of ropivacaine.

In our case, midazolam 2 mg was administrated intravenously by the anesthesiologist 4 min after the commencement of seizures. No additional dose was needed, since the seizures were immediately controlled. It is likely that a great amount of anesthetic agent was rapidly eliminated from the vascular bed leading to cessation of seizures and the administration of midazolam played a minimal role in controlling the seizures.

## Conclusion

In conclusion, we present a case in which generalized tonic-clonic seizures developed during retrobulbar anesthesia of ropivacaine and before completion of the anesthetic injection, followed by transient contralateral hemiparesis. The early onset of seizures indicated intra-arterial injection of the anesthetic. Our case indicated the necessity of needle aspiration before injection for preventing serious complications during local anesthesia. Furthermore, our case suggested the need for a peripheral venous catheter before retrobulbar injection for immediate intravenous access, close monitoring during the performance of the procedure and the presence of well-trained personnel for early recognition and immediate management of the complications.

## Consent

Written informed consent was obtained from the patient for publication of this Case report and any accompanying images. A copy of the written consent is available for review by the Editor of this journal.
